# Optimization of Enzymatic Production of Oligopeptides from Apricot Almonds Meal with Neutrase and N120P

**DOI:** 10.3390/ijms11124952

**Published:** 2010-12-02

**Authors:** Chunyan Wang, Qiang Wang, Jinqiang Tian

**Affiliations:** Institute of Agro-food Science and Technology, The Key Laboratory of Agro-food Process and Quality Control, Ministry of Agriculture, Chinese Academy of Agriculture Science, 2 Yuanmingyuanxi Road, Beijing 100193, China; E-Mails: wchunyan001@163.com (C.W.); tianjinqiang1971@163.com (J.T.)

**Keywords:** apricot almonds meal, enzymatic production, hydrolysis, oligopeptides

## Abstract

Neutrase 0.8L and N120P proteases were used for oligopeptide production from apricot almonds meal, and response surface design was carried out to optimize the effect of hydrolysis conditions on hydrolysis degree (DH) and oligopeptide yield rate. Four independent variables were used to optimize the hydrolysis process: hydrolysis temperature (*X*_1_), enzyme-to substrate ratio (E/S) (*X*_2_), substrate concentration (*X*_3_) and reaction time (*X*_4_). Statistical analysis indicated that the four variables, quadratic terms of *X*_1_, *X*_3_, and *X*_4_, and the interaction terms with X_1_ had a significant (*p* < 0.05) effect on DH. The yield rate was also significantly affected by the four variables and quadratic terms of *X*_1_, *X*_2_ and *X*_4_. Two mathematical models with high determination coefficient were obtained and could be employed to optimize protein hydrolysis. The optimal hydrolysis conditions were determined as follows: hydrolysis temperature 52.5 °C; enzyme-to-substrate ratio (E/S) 7200 U/g; substrate concentration 2%; reaction time 173 min. The initial pH 6.5 and Neutrase-to-N120P dosage ratio 2:1 were fixed in this study according to the preliminary research. Under these conditions, the experimental DH and yield rate were 34.10 ± 5.25% and 72.42 ± 2.27%, respectively.

## 1. Introduction

The apricot is a member of the Rosaceae, subfamily Prunoideae. It is widely grown in Asia, the Mediterranean region and the United States of America. The apricot almond constitutes an important part of the human diet. They are typically used as snack foods or as ingredient in a variety of processed foods, especially in bakery and confectionery products [1,2]. In recent years, apricot kernels are used in the production of oils, benzaldehyde, cosmetics, aroma perfume, and active carbon [3,4]. Almond contains 15–30% protein and its amino acid composition is found to be balanced [5]. Defatted almonds meal (DAM) contains 41.5% total protein and the amount of it remaining after processing is quite large, so almond meal could be used as a good source of protein [5]. The research of almond protein mainly focuses on physico-chemical and functional properties and the protein allergy [6,7]. There are few studies on deep processed dietary protein production in the waste of DAM, and just a small part of it was used to produce livestock feed. Therefore, it is necessary to research how to utilize DAM.

During the last decade, many bioactive peptides have been discovered from enzymatic hydrolysate of different food proteins, including mineral binding peptides, immunomodulatory peptides [8], antibacterial peptides [9], antithrombotic peptides [10], and antihypertensive peptides [11,12]. To our knowledge, most of these peptides are prepared by enzyme hydrolysis; in addition small peptides are better absorbed than proteins and free amino acids [13,14]. Therefore, a high degree of hydrolysis (DH) and high peptide yield rates are desirable.

In the process of hydrolysis, the influence of hydrolysis parameters, including temperature, enzyme-to-substrate (E/S) ratio, substrate concentration and hydrolysis time and the interactive effects between hydrolysis parameters on DH and peptide yield rate have to be considered. In order to optimize hydrolysis conditions and establish predictive models of the effects of various hydrolysis parameters on the DH and yield rate of almond oligopeptides, the hydrolysis process has to be further investigated. Response surface modeling has proven to be an effective statistical technique for optimizing complex processes [15].

The major goal of this study was to use Neutrase 0.8L and N120P to produce oligopeptides from DAM. Also, a study of response surface design was carried out to examine the effect of hydrolysis parameters such as hydrolysis temperature, enzyme-to-substrate (E/S), substrate concentration, hydrolysis time and their interactive effects on DH and oligopeptide yield rate. Furthermore, the predictive models and optimal condition of the hydrolysis process were established through response surface analysis in order to obtain high DH and high oligopeptide yield rate. To our knowledge, there have been little studies on peptides derived from almond protein.

## 2. Results and Discussion

### 2.1. Analysis of Response Surface

**Figure 1.**
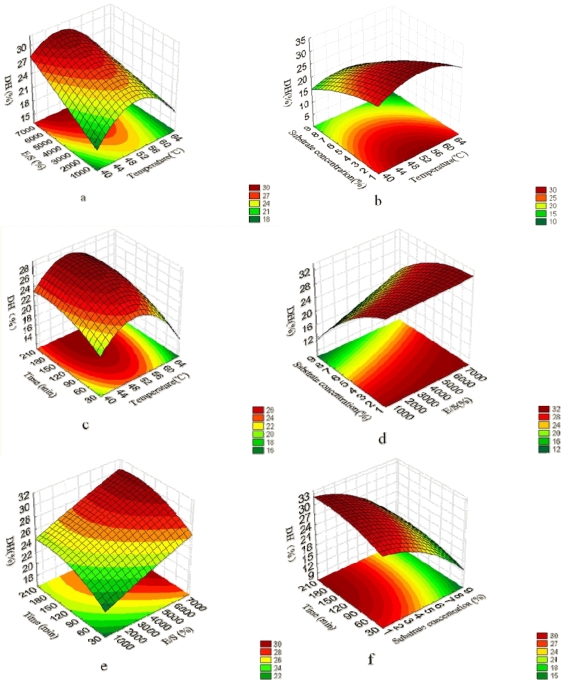
Response surfaces for the interaction of variables on DH. The response surface plots at various (**a**) temperatures and E/S; (**b**) temperatures and substrate concentrations; (**c**) temperatures and reaction times; (**d**) E/S and substrate concentrations; (**e**) E/S and reaction times; and (**f**) substrate concentrations and reaction times.

The relationship between independent and dependent variables is illustrated in tri-dimensional representation of the response surfaces generated by the model for DH (Figure 1). Due to the same trend of variables affecting DH and almond oligopeptide (AOP) yield rate (Equations (1), (2) and Table 3), only the response surface analysis of DH was performed. Two variables were depicted in one tri-dimensional surface plot while the other variables were kept at level zero. With the changing of the variables, the variation of DH was significant. It is clear that the DH was sensitive to alteration of the test variables.

The effect of the interaction relationship of temperature with E/S, substrate concentration and reaction time on DH are shown in Figure 1a–c, respectively, which together indicate that these four variables all significantly affected DH. As shown in Figure 1a–c, the DH enhanced rapidly with an increase in temperature and reached a peak value at 52.5 °C. With further temperature increases, the DH decreased significantly. E/S and reaction time had a positive effect on DH, while the DH decreased when the substrate concentration was in the range of 2–8%. Longer reaction times and higher E/S had positive effects on the yield extraction, and reached a critical value at 173 min and 5851 units g^−1^ protein, respectively, when at a constant temperature (52.5 °C). This suggested the higher DH resulted at a medium temperature, higher E/S, longer reaction time and lower substrate concentration.

It is considered that a higher DH at higher E/S is due to the increase of the contact chance of enzyme and protein and enhanced concentration of peptide bonds susceptible to hydrolysis by the proteases. The same results were obtained by Zhang *et al.* and Wang *et al.* [16,17]. For the substrate concentration, a negative effect on DH was observed due to the higher substrate concentration leading to a decrease in water activity, and the substrate may play a part in deactivating the enzyme at the lower water activity, which is in accordance with other studies [18,19].

The effect of E/S, substrate concentration and reaction time are illustrated in the response surface plots. It is shown that the interactions between the E/S and other two variables did not impact the DH significantly, despite the three variables being the main factors affecting the DH (Table 3, Figure 1d–f). Figure 1d shows the response surface plot at various E/S and substrate concentrations. The DH decreased rapidly with the increasing substrate concentration, while there was less effect on the DH with the increasing of E/S, which further validates there was not interaction between E/S and substrate concentration. The response curves shown in Figure 1e and Figure 1f were comparatively smooth at lower E/S and higher substrate concentration, indicating less effect on increasing the DH when the reaction time changed within the range 30 min to 210 min. This result indicates that no significant interaction existed between E/S and reaction time and between substrate concentration and reaction time. Longer reaction times resulted in higher DH, higher E/S and lower substrate concentrations in the experimental range.

### 2.2. Fitting the Model

The response results shown in Table 1 were analyzed using Statistic 6.0 software. A regression analysis (Table 2) was carried out to fit mathematical models to the experimental data aiming at an optimal region for the responses studied. Predicted response *Y*_1_ for the DH of DAM hydrolysis and *Y*_2_ for the AOP yield rate could be expressed by the following second order polynomial equations in terms of coded values:

(1) $$\begin{array}{l} Y_{1}=26.9838-1.1956X_{1}+1.7267X_{2}-3.6016X_{3}+1.1299X_{4}-1.9914X_{1}^{2}-0.6798X_{3}^{2}\\ -0.5118X_{4}^{2}-0.6538X_{1}X_{2}-0.88125X_{1}X_{3}+0.76875X_{1}X_{4} \end{array}$$

(2) $$\begin{array}{l} Y_{2}=59.1784-1.3525X_{1}+2.2386X_{2}-6.2446X_{3}+2.3572X_{4}-3.1715X_{1}^{2}-1.5010X_{2}^{2}\\ -1.0997X_{4}^{2} \end{array}$$

where *Y*_1_ and *Y*_2_ are the predicted response in real value; *X*_1_, *X*_2_, *X*_3_, *X*_4_ the coded values of temperature, E/S, substrate concentration and reaction time, respectively.

Table 2 presents the analysis of variance (ANOVA) for the fitted quadratic polynomial model of DH and AOP yield rate. The high model *F*-value (*F* = 69.655 and 39.172) and the low *p*-value (*p* < 0001) indicate that the models were highly significant. *R*^2^_adj_ (adjusted determination coefficient) is the correlation measure for testing the goodness-of-fit of the regression equation between experimental and model predicted values. The higher this value, the better the degree of correlation between the observed and predicted values [20]. The value of *R*^2^_adj_ for Equation (1) is 0.9858 and that for Equation (2) is 0.9502, which are reasonably close to 1 and imply that 98.58% of the total variation can be explained by model Equation (1) and 95.02% of the total variations can be explained by model Equation (2). The slope of the correlation shows that most of the actual values were under the prediction of the values. The ANOVA also shows that the lack of fit was non-significant (*p* > 0.05), which further validates the models.

The *t*-test and *p*-value were used to check the effect of each factor on DH and AOP yield rate (Table 3). The data in Table 3 indicate that all the independent variables (*X*_1_, *X*_2_, *X*_3_, *X*_4_) and three quadratic terms (*X*_1_^2^, *X*_3_^2^ and *X*_4_^2^) significantly affected the DH, and there was significant interaction between temperature and the other three variables (E/S, substrate concentration and reaction time). Meanwhile, it can be seen that the AOP yield rate was influenced by all the four independent variables and three quadratic terms (*X*_1_^2^, *X*_2_^2^ and *X*_4_^2^). Interaction between variables did not significantly impact AOP.

### 2.3. Optimization of Hydrolysis Parameters and Validation of the Model

To validate the practicability and veracity of the equation, the experiment was run at optimum conditions within the experimental range obtained from the above study. DH and yield rate were obtained at the optimum level were 34.10 ± 5.25% and 72.42 ± 2.27% (*N* = 3), respectively. This is significantly in agreement with the calculated values (*p* > 0.05). The results of analysis confirmed that the response models were adequate for reflecting the expected optimization, and the model of Equation (1) and Equation (2) were satisfactory and accurate.

## 3. Experimental Section

### 3.1. Materials

Neutrase 0.8L (1.6 × 10^5^ units g^−1^ protein) was purchased from Novozymes A/S (Bagsvaerd DK-2880, Denmark) and N120P (2.2 × 10^5^ units g^−1^ protein) was purchased from Co. Kerry (Prince’s Street, Tralee, Ireland). Commercial defatted almonds meal (DAM), obtained from Aolike Ecogocal Agriculture Co.ltd (Xinjiang, China), was used as hydrolysis action substrate. The chemical composition of DAM was as follows: protein, 51.28%; moisture, 3.5%; lipid, 7.0%; ash, 4.8%; and crude fiber, 7.81% (all data were provided by the supplier). All chemicals used in this investigation were of analytical grade and purchased from Beijing Chemicals Co. (Beijing, China).

### 3.2. Preparation of Apricot Kernel Oligopeptides

In the present study, DAM was hydrolyzed with Neutrase 0.8L which cleaves peptides bonds with broad specificity, produced by *Bacillus amyloliquefaciens*. N120P is a food-grade enzyme produced by *Bacillus subtilis*, which has been shown to hydrolyse peptides with aromatic amino acids and Ala, Val. Because 20% of the amino acids are Ala, Val and Phe, and so on [1,21,22], these proteases can hydrolyse apricot kernel protein effectively. The process of hydrolysing DAM to prepare almond oligopeptides (AOP) was performed in a jacketed glass reactor connected to a thermostatically controlled water heater (CS501-SP, SiDa Science Instruments Inc. Chongqing Province, China) to maintain a constant temperature of suspension for the whole hydrolysis processing. The DAM was mixed with water and the substrate concentration was 2%, 3.2%, 5%, 6.8%, 8%, respectively. Prior to hydrolysis, the DAM solution was stirred for 15 min at the pretreatment temperature of 85 °C. During hydrolysis, enzyme-to-substrate ratio was 550–7,200 units g^−1^ protein. According to our preliminary research, the suspension pH was maintained at 6.5 by the addition of 1 mol L^−1^ NaOH, the enzyme ratio of Neutrase 0.8 to N120P was 2:1. The hydrolysis process was terminated by heating the reactants at 90 °C for 10 min, followed by cooling to room temperature and centrifugation of the suspension at 4200 rpm for 15 min to separate the solid and liquid phases. Finally, the supernatant of hydrolysate were freeze-dried at −40 °C and stored at 20 °C for further use.

The DH, which is defined as the percentage of peptide bonds cleaved by protease, was determined according to the OPA method [23]. The soluble nitrogen was determined by a modified Lowry’s method using bovine serum albumin as a standard [24], and the AOP yield rate was assayed with the methods described by Jang *et al.* [25].

### 3.3. Experimental Design

According to our preliminary experiments, the hydrolysis parameters, including hydrolysis temperature (*X*_1_), enzyme-to-substrate ratio (E/S) (*X*_2_), substrate concentration (*X*_3_) and reaction time (*X*_4_), were optimized as independent variables (*K* = 4), while other related hydrolysis parameters, such as pH and Neutrase-to-N120P dosage ratio, were maintained at the optimum level 6.5 and 2:1, respectively, according to our preliminary research based on DH and yield rate. The two dependent variables to evaluate the effect of hydrolysis were DH (%) (*Y*_1_) and yield rate (%) (*Y*_2_) of AOP. The independent variables were optimized using a central composite rotatable design (CCRD) containing five levels for each independent variable, coded as −1.682, −1, 0, +1, +1.682. The ranges of the independent variables are given in Table 4. Table 1 listed the central composite design consisting of 16 experimental points and seven central designs. Each hydrolysis experiment was run in duplicate.

Experimental data were fitted to a quadratic polynomial model and regression coefficients obtained. The non-linear computer generated quadratic model used in the response surface was of the form:

(3) $$Y=\beta_{0}+\sum_{i=1}^{4}\beta_{i}X_{i}+\sum_{i=1}^{4}\beta_{\text{ii}}X_{i}^{2}+\sum_{i=1}^{4}\beta_{ij}X_{i}X_{j}$$

where *Y* was the predicted response, *β* the intercept term, *β_i_* the linear coefficients, *β_ii_* the quadratic coefficients, *β_ij_* the interactive coefficients, and *X_i_* and *X_j_* the coded independent variables. Data were expressed as means of duplicated determinations. The responses obtained from each set of CCRD experimental design (Table 1) were subjected to multiple non-linear regressions using the Design Expert software (Statistic version 6.0, Statsoft inc., Tulsa, OK, USA). The significance of the equation parameters for each response variable was assessed by the student’s-*t* test. The level of significance was defined at *p* < 0.05. The quality of fit of model was evaluated by the analysis of variance (ANOVA).

## 4. Conclusions

DAM, which was a good substrate to produce peptides, was effectively hydrolyzed by Neutrase 0.8L and N120P proteinases to obtain oligopeptides with a high DH and yield rate. Response surface analysis was an efficient statistical analysis tool in the optimization of the hydrolysis conditions. DH and yield rate was influenced significantly by hydrolysis temperature, E/S, substrate concentration and reaction time (*p* < 0.05). For the model of DH, all the independent variables, quadratic of temperature, substrate concentration and extraction time had highly significant effects on the response values, followed by a significant interaction effect between temperature and the other three variables. For the model of yield rate, all the independent variables, quadratic of temperature, E/S and reaction time significantly affected the response values, and there was no interaction between variables. A high correlation of the quadratic polynomial mathematical model was obtained and could be employed to optimize the hydrolysis of apricot kernel protein meal by Neutrase and N120P proteases. According to the preliminary work and central composite rotatable design in this study, the highest DH and yield rates were 34.10 ± 5.25% and 72.42 ± 2.27%, respectively, at pH 6.5, with a Neutrase-to-N120P dosage ratio 2:1 and hydrolysis temperature 52.5 °C, enzyme-to-substrate ratio (E/S) 7200 units g^−1^ protein, and substrate concentration 2%, reaction time 173 min.

## Figures and Tables

**Table 1.** The factorial central composite design matrix for hydrolysis DH and yield rate: hydrolysis temperature (°C) (*X*_1_), enzyme-to-substrate ratio (E/S) (units g^−1^ protein) (*X*_2_); substrate concentration (%) (*X*_3_), reaction time (min) (*X*_4_) were the four independent variables.

**Trial No.**	**Independent Variables**				**DH (%)**		**Yield Rate (%)**	
	
	*X*_1_	*X*_2_	*X*_3_	*X*_4_	**Actual Value**	**Predicted Value**	**Actual Value**	**Predicted Value**
1	59.9	5852	6.8	174	21.29	21.04	52.54	52.07
2	59.9	5852	3.2	66	27.04	26.21	59.58	59.15
3	59.9	1898	6.8	66	15.07	15.10	41.47	40.08
4	59.9	1898	3.2	174	28.02	27.86	57.54	57.05
5	45.1	5852	6.8	66	24.21	24.24	46.68	47.02
6	45.1	5852	3.2	174	30.56	30.40	63.22	64.46
7	45.1	1898	6.8	174	19.5	20.20	49.32	49.60
8	45.1	1898	3.2	66	24.8	24.92	57.75	58.07
9	40	3875	5	120	23.84	23.36	53.89	52.514
10	65	3875	5	120	18.68	19.34	46.38	47.97
11	52.5	500	5	120	24.4	23.93	50.51	51.20
12	52.5	7200	5	120	29.08	29.74	59.21	58.73
13	52.5	3875	2	120	30.57	31.11	70.32	69.71
14	52.5	3875	8	120	19.37	19.00	48.2	48.71
15	52.5	3875	5	30	23.31	23.64	51.52	52.14
16	52.5	3875	5	210	27.58	27.44	60.47	60.06
17	52.5	3875	5	120	27.49	26.99	58.46	59.21
18	52.5	3875	5	120	27.69	26.98	56.4	59.21
19	52.5	3875	5	120	27.48	26.98	60	59.21
20	52.5	3875	5	120	26.97	26.98	61.03	59.21
21	52.5	3875	5	120	26.49	26.98	59.23	59.21
22	52.5	3875	5	120	26.49	26.98	60	59.21
23	52.5	3875	5	120	26.49	26.98	59.3	59.21

**Table 2.** The analysis of variance (ANOVA) for the fitted quadratic polynomial model of DH and AOP yield rate.

**Source**	**DF**	**SS**	**MS**	***F*-value**	**Prob > *F***
DH (%)					
Residual	11	343.122	31.193	69.655	0.0001**
Lack of fit	11	4.926	0.448		
Pure error	5	3.199	0.639	2.22405	0.125
Cor total	6	1.7264	0.288		
Regression	22	348.048			
		*R*^2^ = 0.9859	*R*^2^_Adj_ = 0.9858		
Yield rate (%)					
Residual	11	928.407	84.401	39.172	0.0001**
Lack of fit	11	23.701	2.1546		
Pure error	5	10.674	2.135	0.983	0.469
Cor total	6	13.027	2.171		
Regression	22	952.108			
		*R*^2^ = 0.9751	*R*^2^_Adj_ = 0.9502		

** significant at 0.01.

**Table 3.** Significance of regression equation coefficients for the DH and AOP yield rate.

**Variable**	**DH**				**Yield Rate**			

	**Regression coefficients**	**Standard error**	***t*-value**	***p*-value**	**Regression coefficients**	**Standar *d* error**	***t*-value**	***p*-value**
*X*_1_	−0.8936	0.1810	6.6025	0.0002	−0.7163	0.3971	3.4050	0.0143
*X*_2_	0.9445	0.1678	9.5353	0.0001	0.8618	0.3682	5.6358	0.0005
*X*_3_	−0.9864	0.1810	19.8892	0.0001	−0.9785	0.3971	15.7214	0.0001
*X*_4_	0.8830	0.1678	6.2399	0.0002	0.8729	0.3682	5.9345	0.0004
*X*_1_*X*_1_	−0.9631	0.1810	11.8620	0.0001	−0.9332	0.3971	8.6124	0.0001
*X*_2_*X*_2_	−0.0965	0.1678	0.3214	0.7891	−0.7757	0.3682	4.0760	0.0052
*X*_3_*X*_3_	−0.7736	0.1810	4.0490	0.0054	0.0447	0.3971	0.1484	0.9016
*X*_4_*X*_4_	−0.6767	0.1678	3.0486	0.0247	−0.6691	0.3682	2.9863	0.0272
*X*_1_*X*_2_	−0.6401	0.2365	2.7632	0.0381	0.5982	0.5189	2.4761	0.0584
*X*_1_*X*_3_	−0.7468	0.2365	3.7247	0.0088	0.1339	0.5189	0.4480	0.7097
*X*_1_*X*_4_	0.6998	0.2365	3.2492	0.0182	0.0667	0.5189	0.2216	0.8536

**Table 4.** Coded settings of the independent variables for DAM hydrolysis, according to central composite rotatable design: hydrolysis temperature (°C) (*X*_1_), enzyme-to-substrate ratio (E/S) (units g^−1^ protein) (*X*_2_); substrate concentration (%) (*X*_3_), reaction time (min) (*X*_4_) were the four independent variables.

**Coded Level**	**Independent Variables**			
	***X*_1_ (°C)**	***X*_2_ (units g^−1^ protein)**	***X*_3_ (%)**	***X*_4_ (min)**
1.682(+γ)	65	7200	8	210
1	59.9	5852	6.8	174
0	52.5	3875	5	120
−1	45.1	1898	3.2	66
−1.682(−γ)	40	550	2	30
